# ATX-101, a Peptide Targeting PCNA, Has Antitumor Efficacy Alone or in Combination with Radiotherapy in Murine Models of Human Glioblastoma

**DOI:** 10.3390/cancers14020289

**Published:** 2022-01-07

**Authors:** Giovanni Luca Gravina, Alessandro Colapietro, Andrea Mancini, Alessandra Rossetti, Stefano Martellucci, Luca Ventura, Martina Di Franco, Francesco Marampon, Vincenzo Mattei, Leda Assunta Biordi, Marit Otterlei, Claudio Festuccia

**Affiliations:** 1 Department of Biotechnological and Applied Clinical Sciences, Division of Radiation Oncology, University of L’Aquila, 67100 L’Aquila, Italy; giovanniluca.gravina@univaq.it; 2 Department of Biotechnological and Applied Clinical Sciences, Laboratory of Radiobiology, University of L’Aquila, 67100 L’Aquila, Italy; alecolapietro@gmail.com (A.C.); mancio_1982@hotmail.com (A.M.); alessandra.rossetti@graduateunivaq.it (A.R.); 3 Department of Biotechnological and Applied Clinical Sciences, Laboratory of Cellular Pathology, University of L’Aquila, 67100 L’Aquila, Italy; s.martellucci@sabinauniversitas.it; 4 Biomedicine and Advanced Technologies Rieti Center, Sabina Universitas, 02100 Rieti, Italy; vincenzo.mattei@uniroma1.it; 5 Division of Pathology, San Salvatore Hospital, 67100 L’Aquila, Italy; lventura@asl1abruzzo.it (L.V.); mdifranco@asl1abruzzo.it (M.D.F.); 6 Department of Radiological, Oncological and Pathological Sciences, Sapienza University of Rome, 00100 Rome, Italy; francesco.marampon@uniroma1.it; 7 Department of Biotechnological and Applied Clinical Sciences, Laboratory of Medical Oncology, University of L’Aquila, 67100 L’Aquila, Italy; Leda.biordi@univaq.it; 8 APIM Therapeutics A/S, N-7100 Rissa, Norway; 9 Department of Clinical and Molecular Medicine, Norwegian University of Science and Technology (NTNU), N-7006 Trondheim, Norway

**Keywords:** GBM, DNA damage signaling, APIM-peptide

## Abstract

**Simple Summary:**

PCNA is an interesting target for cancertreatment due to its essential activities in DNA replication and repair and its recently discovered regulatory roles in cellular signaling. Here, we demonstrate that ATX-101, a peptide targeting PCNA, has antitumor effects as a single agent and radiosensitizing properties in glioblastoma multiforme models.

**Abstract:**

Cell proliferation requires the orchestrated actions of a myriad of proteins regulating DNA replication, DNA repair and damage tolerance, and cell cycle. Proliferating cell nuclear antigen (PCNA) is a master regulator which interacts with multiple proteins functioning in these processes, and this makes PCNA an attractive target in anticancer therapies. Here, we show that a cell-penetrating peptide containing the AlkB homolog 2 PCNA-interacting motif (APIM), ATX-101, has antitumor activity in a panel of human glioblastoma multiforme (GBM) cell lines and patient-derived glioma-initiating cells (GICs). Their sensitivity to ATX-101 was not related to cellular levels of PCNA, or p53, PTEN, or MGMT status. However, ATX-101 reduced Akt/mTOR and DNA-PKcs signaling, and a correlation between high Akt activation and sensitivity for ATX-101 was found. ATX-101 increased the levels of γH2AX, DNA fragmentation, and apoptosis when combined with radiotherapy (RT). In line with the in vitro results, ATX-101 strongly reduced tumor growth in two subcutaneous xenografts and two orthotopic GBM models, both as a single agent and in combination with RT. The ability of ATX-101 to sensitize cells to RT is promising for further development of this compound for use in GBM.

## 1. Introduction

Glioblastoma multiforme (GBM) is a very aggressive brain tumor [1] characterized by high inter- and intratumor heterogeneity [2] with high local invasiveness [3], extensive necrosis [4], and high vascularity [5]. Current standard treatments for this neoplasm include surgical resection combined with radiotherapy (RT) and chemotherapy [6]. Despite advances and improvements of this therapeutic approach, patient outcomes remain poor. Therefore, the development of new and effective therapies is in high demand.

Greater knowledge of the molecular traits of GBM, i.e., altered cell signaling, can lead to more efficient targeted therapeutic approaches [7]. However, so far, targeted therapies have shown only limited efficacy in patients [8]. RT is an important treatment modality for GBM [9], alone or in association with temozolomide [4]. This therapy can induce various types of cell death [10], including apoptosis, necrosis, necroptosis, and autophagy, and RT may have both immunosuppressive and immunogenic effects. It has been well established that tumor cells that initially show a good therapeutic response to RT and later often develop resistance to treatment. Differential radiosensitivity and induction of radioresistance have been associated with altered responses to DNA damage and repair of DNA double-strand breaks (DSBs) [11,12]. Among other factors, particular hypoxia has an important impact on RT resistance [13,14,15,16]. Recent approaches to improve the sensitivity of RT and chemotherapy have therefore focused on targeting key proteins involved in DNA repair [17], DNA damage checkpoint activation [18], and hypoxia signaling [19,20]. However, the resistance of GBM to RT, as well as temozolomide treatment, is still the main reason for therapy failure [21].

Tumors, including GBMs, are represented by a heterogeneous cell population consisting of cells with different potentials for proliferation. Progeny cells consequently establish a hierarchical organization of stem cells, transient amplifying cells, and terminally differentiated cells. Glioma-initiating cells (GICs) showing a glioma stem cell (GSC) phenotype [22,23] colonize tumor areas with elevated hypoxia and “palisading necrosis” [24,25]. Residual infiltrating GICs result in less sensitivity to therapies and consequently GBM recurrence [26,27]. Moreover, resident nontumor cells, including infiltrating lymphocytes and glioma-associated microglia/macrophages constituting the tumor stroma [28,29], are responsible, in part, for the altered malignancy of GBM, as this cell population supports angiogenesis, growth, and invasiveness.

Proliferating nuclear antigen (PCNA) is a scaffold protein belonging to the functionally and structurally conserved DNA sliding clamp family of proteins, which is essential for DNA replication, DNA repair and damage tolerance, and chromatin remodeling [30]. However, recently, PCNA has also been found to be involved in regulation of apoptosis and cellular signaling [31,32,33]. PCNA may potentially interact with more than 600 proteins through either of the two PCNA-interacting motifs: the PCNA-interacting peptide (PIP)-box and APIM [34,35]. The PIP-box and APIM have overlapping interaction sites on PCNA [32,36], but while most of the essential replicative proteins interact with PCNA through the high-affinity canonical PIP-box, proteins involved in stress management often contain APIM or noncanonical PIP-boxes, which have lower affinities for PCNA in the absence of stress. During cellular stress, PCNA is post-translationally modified, and the proteins containing the low-affinity PCNA-interacting motifs often have additional binding motifs for these posttranslational modifications, e.g., mono- or polyubiquitin, which increases their affinity for PCNA [37]. Multiple proteins involved in cell signaling contain a putative PCNA-interacting motif, e.g., several APIM-containing proteins are involved in the MAPK and PI3K/Akt pathways acting downstream of multiple receptor tyrosine kinases (RTKs) [33]. Accordingly, targeting PCNA with a cell-penetrating peptide containing APIM (APIM-peptide) is shown to mediate changes in PI3K/Akt and MAPK pathways and increase apoptosis in cancer cells also in the absence of exogenous DNA damage [32,33,38]. This is likely due to enhanced levels of endogenous stress in cancers.

The cytotoxic effects of RT are mediated mainly by the induction of single- or double-stranded DNA breaks (SSBs or DSBs) and by generations of reactive oxygen species (ROS). After the introduction of DSBs, histone H2AX is rapidly phosphorylated (γH2AX), marking the damaged area for DNA repair [39]. Given the central role of PCNA in DNA repair, targeting PCNA, protein interactions may represent a good strategy to increase RT sensitivity similar to what has been observed for chemotherapeutics when combined with the APIM-peptide [40,41,42,43]. An experimental APIM-peptide drug, ATX-101, was recently tested in a Phase I study in cancer patients (cancer patients with advanced solid tumors, all-comers) [44]. ATX-101 was shown to have a favorable toxicity profile (no myeloid-suppression) and a cancer-stabilizing effect, supporting that ATX-101 mainly targets modified PCNA in stressed cells and does not block normal replication [32,40]. Here, we examined the antitumor activity of ATX-101 in a panel of human GBM cell lines and GICs in vitro and in vivo in combination with RT. We found that ATX-101 has anticancer activity as a single agent in vitro and in vivo and a good RT-sensitizing effect in both subcutaneous and intracranial xenograft tumor models. Our data suggest that targeting the stress regulatory mechanisms of PCNA may be a good strategy to increase RT efficacy meriting further examinations.

## 2. Materials and Methods

### 2.1. Reagents, Antibodies, and Drug Preparation

Materials for tissue cultures were purchased from Euroclone (Milan, Italy). Ki67 antibody was purchased from Dako (Carpenteria, CA, USA). Antibodies for anti-active/cleaved caspase-3 (ab32042), caspase-9 (ab2324), and GAP43 (ab75810) were purchased from Abcam (Cambridge Biomedical Campus, Cambridge, UK). The antibodies against phospho-Akt (S473) (STJ90166), phospho-Akt (T308) (STJ90167), total Akt (STJ91538), total 4E-BP1 (STJ91385), phospho-4E-BP1(S65) (STJ90778), and CD44 (STJ193181) were purchased from Saint John’s Laboratory (Docklands Campus University Way, London, UK). Antibodies against β-actin (sc-69879), PCNA (sc-56), p-Histone H2AX (Ser 139) (γH2A, sc-517348), DNA-PKcs (sc-390849), GFAP (sc-3673), Oct-3/4 (sc-5279), β3 Tubulin (sc-80005), NFH (sc-133237), and Sox2 (sc-365823) were purchased from Santa Cruz (Santa Cruz, CA, USA). Antihuman CD34 antibody (BioLegend, London, UK) was used in IHC. Secondary antibodies for Western blots were purchased from Santa Cruz. Antibodies were used at dilutions according to the manufacturer’s instructions. ATX-101 was provided by APIM Therapeutics A/S, Trondheim, Norway. ATX-101 was dissolved in DMSO (1.0 μM). Aliquots were stored at –20 °C and the stock “in-use” aliquots at 4 °C. The vehicle received the same amount of DMSO compared to the stock. Dilutions were made in serum-free media or buffers and added directly to the cells in the viability assay. For in vivo use, ATX-101 was diluted as described below and injected intraperitoneally (i.p.).

### 2.2. Cell Lines

Twelve human glioma cell lines (U251, U373, U118, U138, A172, U87MG, SW1783, SNB19, LN229, T98G, SF268, and D54) were purchased from ATCC or DSMZ cell collections and were maintained in Dulbecco’s modified Eagle medium (DMEM) containing 10% FBS, 4 mM glutamine, 100 IU/mL penicillin, and 100 μg/mL streptomycin. To minimize the risk of working with misidentified and/or contaminated cell lines, cells were stocked at very low passages. However, periodically, DNA profiling was carried out by using the AmpFLSTRTM identifillerTM PCR amplification kit from Thermo Fisher following the manufacturer’s instructions. Luciferase-transfected U87MG cells were kindly provided by J.E. Heikkila (Abo Akademi University, Turku, Finland). Three GBM patient-derived stem cell lines (BT12M, BT48EF, BT50EF) were kindly provided by J.G. Cairncross and S. Weiss (Arnie Charbonneau Cancer Institute, University of Calgary, Canada) [45,46], and GSCs-5 and GSCs-7 [47,48] were from M. Izquierdo (Universidad Autónoma de Madrid, Spain). Luciferase was inserted in the genome of GSCs-5 cells using the pGL4.13 vector (Promega, Milan, Italy) and the jetPEI DNA transfection method (Polyplus, Illkirch, France). Molecular characteristics of the 12 cell lines used in this report are summarized in Supplementary Table S1.

### 2.3. Cell Viability, Cell Cycle, and Apoptosis Analyses

ATX-101 efficacy was evaluated by using the Cell Counting Kit-8 (CCK-8; Dojindo Molecular Technologies Inc., Tokyo, Japan) and calculating IC50, IC20, and IC10 values by a combination of Grafit software (Erithacus, Wilmington House, UK) and the GR calculator according to the method of Clark et al. [49]. Neurosphere growth was verified by direct count of the spheres originating from single-cell suspensions at different times as described [22,50]. Data on cell cycle and apoptosis were collected by using propidium iodide (PI) and the Alexa Fluor^®^ 488 Annexin V/Dead Cell Apoptosis Kit (Life Technologies Europe BV, Monza, Italy). Annexin is represented in the fluorescence line 1 (FL1) dot plot and PI in FL2. Apoptosis was also quantified by microplate TUNEL assay as described by the manufacturer (TiterTACS™ Colorimetric Apoptosis Detection Kit) from Trevigen (Gaithersburg, MD, USA).

For clonogenic assay, cells were seeded in 6-well plates at a density of 1 × 10^3^ cells per well and were allowed to attach to the plate overnight prior to treatment. Crystal violet was used to evaluate the antiproliferative effects as short-term 24–72 h (h) or long-term (15, 21, or 30 days, depending on the growth rate of considered cells) culture according to Cold Spring Harb Protocol 2016 [51]. Assay of caspases 3, 8, and 9 activities was determined using specific caspase substrates (Kaneka Eurogentec SA, Serain, Belgium). In particular, we used Ac-DEVD-pNA cleaved by caspase-3, Ac—IETD [52]—pNA cleaved by caspase-8 [53], or Ac-Leu-Glu-His-Asp-pNA [54] cleaved by caspase-9. Release of the nitroanilide (pNA) was measured at an optical density (OD) of 450 nm (OD450) in 96-well plates using the Sunrise™ colorimetric plate reader (Tecan Group, Ltd., Männedorf, Switzerland).

### 2.4. Cell Lysates, Western Blot Analysis and Akt/mTOR Enzymatic Analysis

GBM cells were grown in 90 mm diameter Petri dishes at a density of 1 × 10^5^ cells/mL and treated with different doses of ATX-101 (1.0, 2.5, 5, and 10 μM). After 8 h, cells were washed with cold PBS and immediately lysed with 1 mL of lysis buffer containing a proteinase and phosphatase inhibitor cocktail purchased from Cell Signaling Technology (Euroclone, Milan, Italy). Total cell lysates were electrophoresed in 7% or 10% SDS-PAGE. Separated proteins were transferred to nitrocellulose and probed with the appropriate antibodies using the conditions recommended by the suppliers. Protein levels were normalized against GAPDH or β-actin. As previously described [55], we also used in-Cell ELISA assays (Abcam), an immune-cytochemistry method used to quantify target protein or post-translational modifications of the target protein directly in cultured cells, for the evaluation of p-Ser 473-Akt, p-Thr 308-Akt, and p-Ser 65-4E-BP1 activity. OD450 nm was measured using a TECAN Elisa reader (Tecan Italia, Cernusco sul Naviglio, Italy), and the assay was performed according to the protocol recommended by the manufacturer.

### 2.5. Radiation Exposure

Radiation was performed at room temperature using an x-6 MV photon linear accelerator, as previously described [56]. The total single dose of 4 Gy was delivered with a dose rate of 2 Gy/min using a source-to-surface distance (SSD) of 100 cm. A Perspex plate of thickness 1.2 cm was positioned below the cell culture flasks to compensate for the build-up effect. Tumor cells were irradiated placing the gantry angle at 180°. Nonirradiated controls were handled identically except for the radiation exposure. The absorbed dose was measured using a Duplex dosimeter (PTW). For the clonogenic survival assay, exponentially growing GBM cells were harvested and diluted to appropriate densities and plated in triplicate in 6-well plates with 2 mL of complete medium/well in the presence or absence of ATX-101 or vehicle for 24 h (final DMSO concentration of 0.1%, which did not affect the proliferation). After incubation for 24 h, the cells were exposed at room temperature to various doses of radiation. The cells were then washed with PBS, cultured in a drug-free medium for 14 days, fixed with methanol/acetic acid (10:1, *v/v*), and stained with crystal violet. Colonies containing >50 cells were counted. The plating efficiency (PE) was defined as the number of colonies observed/the number of cells plated, and the surviving fraction was calculated as (colonies counted/cells seeded) × (PE/100) [56,57,58].

### 2.6. Drug Enhancement Factor (DEF)

This parameter, also known as radiation dose enhancement [59,60], represents the ratio between the effect of a dose of radiation in combination with an experimental drug relative to the effect observed without. The mean inactivation dose for each treatment was determined, and DEF was calculated as described by Morgan [60]. A DEF value significantly >1 indicates radiosensitization.

### 2.7. Immunofluorescence (IF) and Immuno-Cytochemical (ICC) Analysis

GBM cell lines and GICs were used for IF and ICC analyses. For analyses of GICs, spheres were seeded on glass coverslips pretreated with poly-l-lysine 30 µg/mL, fixed with 4% paraformaldehyde for 20 min at room temperature, permeabilized with 0.3% Triton-X-100 for 5 min at room temperature, and finally incubated overnight at 4 °C with the following primary antibodies according to their datasheets: anti-OCT3/4, Ki67, βIII tubulin, NFH, GFAP, Sox2, Stro-1, CD90, CD44, PCNA, and γH2AX. Adherent GBM cell lines were seeded at 1 × 10^4^ cells/well in 8-well chamber slides (Thermo Fisher, Waltham, MA, USA) and incubated for 24 h prior to treatment. After treatment, the cells were fixed with 100% methanol, washed with PBS cells, and incubated for 30 min at room temperature with AlexaFluor 488 anti-rabbit IgG, AlexaFluor 595 anti-goat IgG, or AlexaFluor 633 anti-mouse IgG secondary antibody (1:2000 Molecular Probes, Invitrogen, Carlsbad, CA, USA). Coverslips were mounted with Vectashield Mounting Medium containing DAPI (0.5 μg/mL) and examined on the confocal microscope (Leica TCS SP5, Mannheim, Germany). The DAB solution was used for the visualization of ICC. The slides were counter stained with hematoxylin solution for 5 min. The slides were finally mounted and visualized by an optical microscope from Zeiss, and images were electronically collected. Quantification of fluorescence was measured using ImageJ Fiji software. The histogram represents the percentage of corrected total cell fluorescence (CTCF) (sample integrated density—background integrated density, arbitrary units (AU)). Integrated density is collected from the area of single selected cells × mean fluorescence of background.

### 2.8. FACS Analyses

Expression of CD44, CD90, GAP43, and βIII tubulin on cells untreated or treated with different doses of ATX-101 was quantified by flow cytometry after paraformaldehyde fixation (4% in PBS) and permeabilization. Washed cells were incubated for 1 h at 4 °C with selected primary antibodies followed by Cy5-conjugated anti-rabbit IgG H&L or PE-conjugated anti-mouse IgG purchased from Abcam for an additional 30 min. All samples were analyzed by using the “BD Accuri™ C6 Plus” flow cytometer (Becton Dickinson Italia Spa, Milan, Italy) equipped with a blue laser (488 nm) and a red laser (640 nm). At least 10,000 events were acquired.

### 2.9. Subcutaneous Xenograft Model

After 1 week of quarantine, female CD1-nu/nu mice, 6 weeks of age (purchased from Charles River, Milan, Italy) and followed under the guidelines established by our Institution (University of L’Aquila, Medical School and Science and Technology School Board Regulations, complying with the Italian government regulation n.116 27 January 1992 for the use of laboratory animals, code 555/2017-PR (CE5C5 01-4-2017) approved 7 July 2017), received two subcutaneous flank injections of 1 × 10^6^ U87MG and T98G cells. The determination of sample size/number of tumors to get a study power of at least 80% (type 1 beta error) was carried out using the MedCalc^®^ statistical software. Next, animals were randomly divided into four different groups containing 5 animals/group. Consequently, 10 (2 × 5) subcutaneous tumors of 0.8–1.3 cm^3^ were treated per group. Treatment was as follows: Group I: Control (vehicle, 200 μL PBS containing 0.9% NaCl), i.p. Group II: ATX-101 in 200 μL PBS containing 0.9% NaCl (8 mg/Kg, 2 days each week at defined times D2, D5, D9, D12, D16, D19, D23, D26, D30, and D33, i.p.; Group III: RT (4 Gy) as single administration at D3; Group IV ATX-101 plus RT. Tumor growth was assessed biweekly by measuring tumor diameters with a Vernier caliper using the formula ‘TW (mg) = tumor volume (mm^3^) = 4/3πR1 × R2 × R3, in which R1/R2/R3 are the 1/2 diameters, thickness/height, length, and width of tumors. To reduce the probability of bias due to differences in tumor engraftment, tumor in progression was defined here as the length of time from the date of randomization or the start of treatments necessary to double the tumor volume for each tumor. The percentage of tumors in progression was plotted over time by using the Kaplan–Meier curve as previously described [50,55,61,62].

### 2.10. Orthotopic Intracranial Model

Female CD1 nu/nu mice were inoculated intracerebrally as previously described [50,55,61,62] with luciferase-transfected U87MG and GSCs-5 cells. Five days after inoculation, when no luciferase activity was intracranially detectable, animals were randomized in 10 mice per group as indicated in Supplementary Table S2 as follows: Group I: Control (vehicle, 200 μL of PBS containing 0.9% NaCl), i.p.; Group II: ATX-101 in 200 μL PBS containing 0.9% NaCl (8 mg/kg, 2 days each week at defined times starting at D5, D8, D12, D15, D19, D22, D26, D29, and D33, i.p.; Group III: RT (4 Gy) in single administration at D7; Group IV ATX-101 plus RT. Primary endpoint was on D36, but the mice were followed for up to 250 days. Tumor growth was assessed every 5th day for bioluminescence intensity (BLI) using the UVITEC Cambridge Mini HD6 (UVItec Limited, Cambridge, United Kingdom). Animals were anesthetized, and luciferin (150 mg/kg) was injected intraperitoneally 15 min prior to imaging. To simulate pathological conditions at the time for which surgery occurs (low number of tumor cells in wound bed causing regrowth and recurrence), we inoculated a small number of cells (1 × 10^3^) and started drug administration before bioluminescence was detected. Mice were euthanized when they displayed neurological signs or weight loss of 20% or greater or when animals bearing tumors died. The time necessary to determine these effects was defined as overall survival (OS), whereas the term of disease-free survival (DSF) defines the time interval in which no bioluminescent signals were shown.

### 2.11. Statistics

Numeric data are expressed as mean ± SD or median with 95% confidence interval (CI). ANOVA was followed by Tukey’s test. TTP, DFS, and OS were analyzed by Kaplan-Meier curves and Gehan’s generalized Wilcoxon test. Values of *p* < 0.05 were considered statistically significant. MedCalc (statistical analysis software package) version 10.0 was used for statistical analysis and graphic presentation.

## 3. Results

### 3.1. ATX-101 Inhibits Glioblastoma Cell Viability

**Figure 1:**
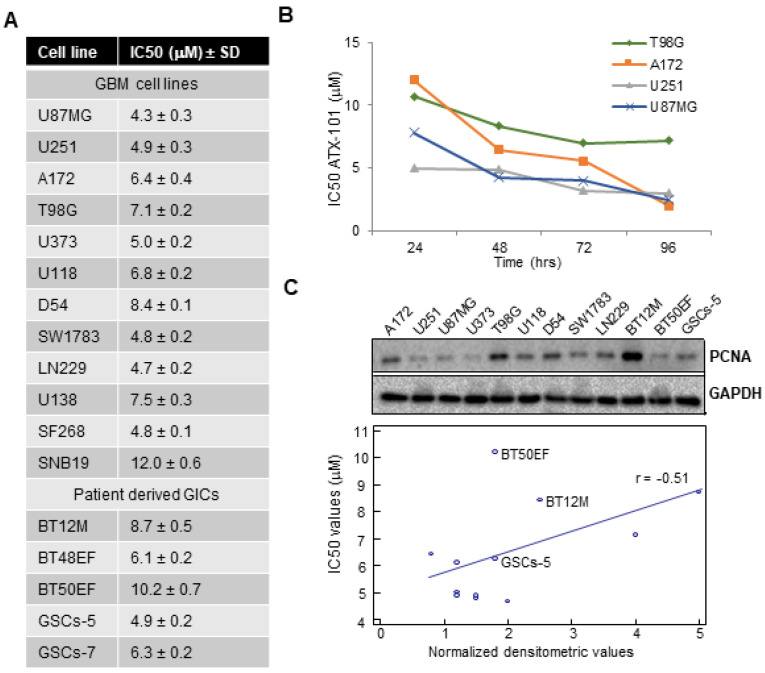
ATX-101 inhibits cell proliferation of GBM and GICs cells in vitro. (**A**) Twelve GBM cell lines and 5 GICs were examined for viability after treatment with ATX-101 (0.1–10 μM). For the five GICs cell lines, the evaluation of cell proliferation was made by using sphere formation assay at clonal growth analyzed after 21 days. IC50 values are calculated for 12 GBM cell lines and 5 GICs at 72 h. (**B**) IC50 values for ATX-101 determined for 4 GBM cell lines over 96 h. (**C**) Upper panel: Western blots showing PCNA and GAPDH expression levels in 9 GBM and 3 GICs cell lines. Uncropped Western blots are shown in Supplementary Figure S2. In the lower panel, PCNA levels normalized against GAPDH were plotted against IC50s. GICs are marked in the graph and the r value (−0.51) is depicted in the graph.

Because human GBMs are extremely heterogeneous, the antiproliferative effects of ATX-101 in a cohort of 12 GBM cell lines and 5 patient-derived GICs showing different molecular assets and sensitivity to standard chemotherapy and RT (Supplementary Table S1) was examined. We observed that GBM cell lines were responsive to ATX-101 with IC50 values between 4.3 (U87MG) and 12.0 μM (SNB19) (Figure 1A). IC50 values of GICs ranged between 4.9 (GSCs-5) and 10.2 μM (BT50EF cells), i.e., not different from the GBM cell lines (Figure 1A, statistics in Supplementary Figure S1C). The effect of ATX-101 increased with time (Figure 1B, exemplified by T98G, A172, U251, and U87MG). Representative inhibition curves with their IC50 determinations for seven cell lines, together with phase-contrast images showing altered morphology in U251 cells and inhibition of colony sphere formation of GSCs-5 (GIC), are shown in Supplementary Figure S1.

When analyzing the expression level of PCNA in total cells extracts from nine GBM and three GIC cell lines, two of the GICs had higher PCNA levels than the GBM cell lines (GICs marked in Figure 1C); however, linear regression of PCNA levels plotted against IC50s did not suggest a correlation between PCNA levels and ATX-101 sensitivity (Figure 1C, r = −0.51)). This is in agreement with previous results [42]. In addition, no correlations were found between high and low IC50s and the function/expression of MGMT, p53, and PTEN (Supplementary Figure S1C).

### 3.2. ATX-101 Inhibits PCNA Expression, Increase the Fraction of Cells in S and G2/M and Induces Apoptosis

**Figure 2:**
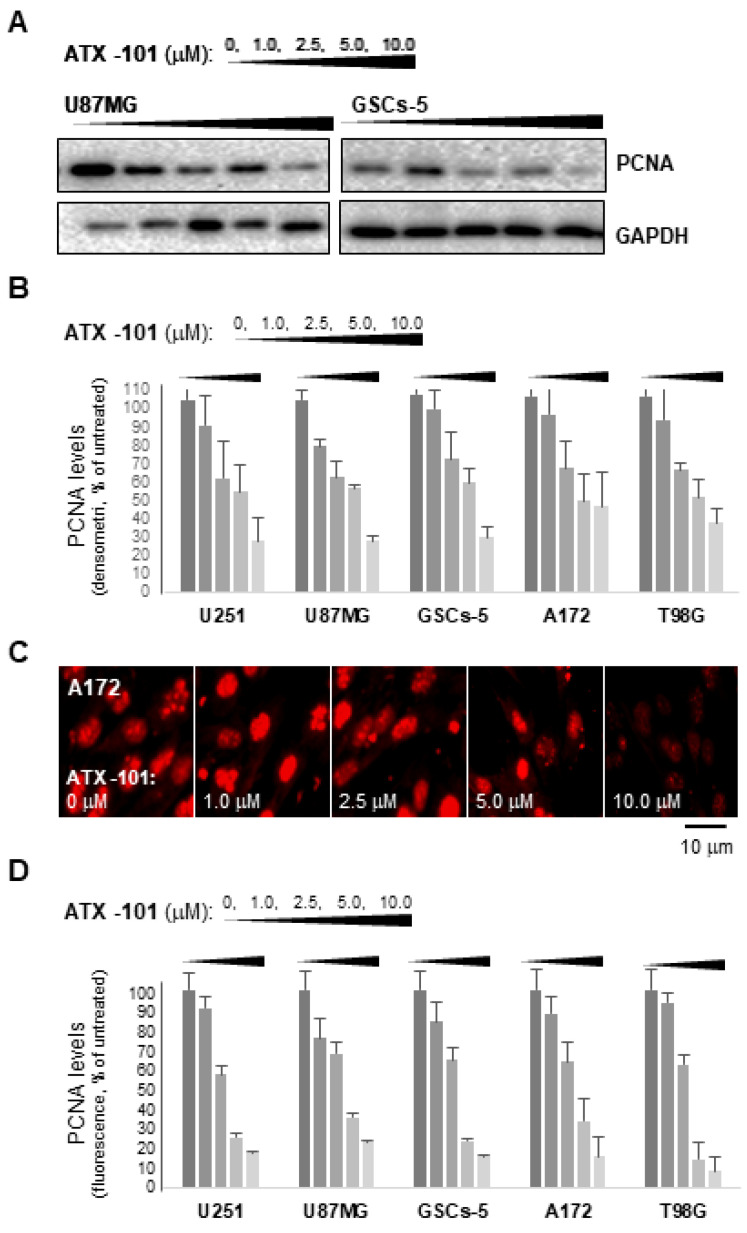
ATX-101 inhibits total PCNA expression in GBM cell models. (**A**) Representative Western blots performed on cell lysates collected from U87MG and GSCs-5 cells treated with ATX-101 (1–10 μM) for 24 h. (**B**) Quantifications of PCNA levels (normalized to GAPDH) in U251, U87MG, GSCs-5, A172, and T98G after treatment with ATX-101 (1–10 μM) for 24 h. Uncropped Western blots are shown in Supplementary Figure S3. (**C**) Immunofluorescence staining of PCNA in A172 cells treated with ATX-101 (1–10 μM) for 24 h. Magnification, ×50; bar corresponds to 10 μm. (**D**) Quantifications of fluorescence in A172 in ATX-101 treated cells (1.0, 2.5, 5.0, 10.0 μM) and in similarly treated U251, U87MG, GSCs-5, and T98G cells. Data presented are the mean ± S.D.

When examining if ATX-101 affected PCNA expression and/or its nuclear distribution in four GBM cell lines and one GIC cell line, it was found that ATX-101 dose dependently inhibited the expression of total PCNA protein at doses below IC50 values in all the cell lines examined (Figure 2A,B; U251 (IC50 = 4.9), U87MG (IC50 = 4.3), GSCs-5 (IC50 = 4.9), A172 (IC50 = 6.4), and T98G (IC50 = 7.1)). Immunofluorescence analyses confirm a reduction of PCNA levels in both the cytoplasm and in the nucleus upon ATX-101 treatment (Figure 2C,D).

**Figure 3:**
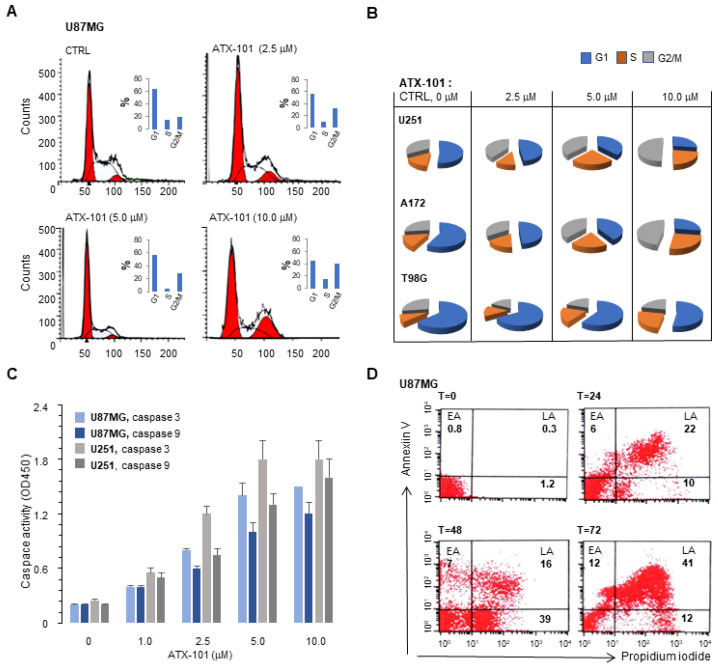
ATX-101 induces G2/M cell arrest and mediates apoptosis. (**A**) Cell cycle analysis of U87MG cells after treatment with ATX-101 (2.5–10 μM) for 24 h. Diploid G1 and diploid G2 cells are marked in red. (**B**) Graphic presentations of cell cycle analysis as in A for U251, A172, and T98G cells. (**C**) Enzymatic caspase 9 and 3 activity in U87MG and U251 cells treated with ATX-101 (2.5–10 μM) for 24 h. Means ± SD of three independent experiments analyzed in triplicates. (**D**) Annexin V and necrosis (by propidium iodide) FACS analyses of U87MG at different time points (T = hours) after addition of 2.5 μM ATX-101. EA = early apoptosis. LA = late apoptosis.

ATX-101 treatment was also found to affect the cell cycle distribution, i.e., an increased number of cells in S and G2/M were found in the four cell lines U87MG, U251, A172, and T98G (Figure 3A,B). This is likely due to elevated replication stress levels in these cell lines because several proteins involved in DNA damage tolerance and replicative stress bind to PCNA via APIM and are likely inhibited by ATX-101 [37,63,64]. Enzymatic analyses of caspase 3 and 9 in U87MG and U251 (Figure 3C) and annexin V/propidium iodide staining of U87MG (Figure 3D) after ATX-101 treatment showed that ATX-101 efficiently induced caspase-3/9-dependent apoptosis, which is in agreement with previous results [32].

### 3.3. ATX-101 Inhibits Akt/mTOR Signaling in GBM Cells

**Figure 4:**
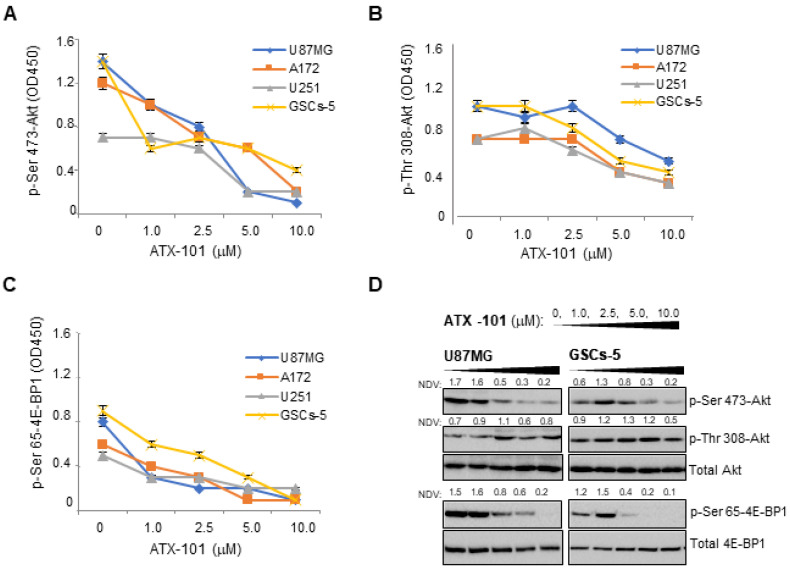
ATX-101 inhibits Akt activity in glioma cells. (**A**–**C**) U87MG, A172, U251, and GSCs-5 cells were treated with ATX-101 (1–10 μM) for 8 h, fixed, and incubated with phospho-specific antibodies in an in-Cell ELISA; (**A**) p-Ser 473-Akt; (**B**) p-Thr 308-Akt; (**C**) p-Ser 65-4E-BP1. OD450 were collected from 5 replicates and given as mean ± SD. (**D**) Extracts from U87MG and GSCs-5 cells treated with ATX-101 (1–10 μM) for 8 h analyzed for p-Ser 473-Akt, p-Thr 308-Akt, total Akt, p-Ser 65-4E-BP1, and total 4E-BPI by Western blot analysis. One representative out of 3 separated experiments with similar results is shown. Uncropped Western blots are shown in Supplementary Figure S4. NDV: Normalization of densitometric values of phosphorylated Akt and 4E-BP1 to nonphosphorylated isoforms are shown above the individual lanes.

Targeting PCNA with ATX-101 was previously shown to reduce Akt signaling in human monocytes [33] and in bladder cancer cells [41]. Here, we examined the effect of ATX-101 treatment on Akt activation in GBM cells through detection of phosphorylated Ser 473 and Thr 308 (p-Ser 473-Akt, p-Thr 308-Akt). For these analyses, we used three PTEN-deficient cell lines with elevated basal levels of Akt activity, U87MG, U251, and A172, and the GSCs-5 cell line, where the differentiation status is dependent on the Akt/mTOR pathway. ATX-101 dose dependently inhibited the phosphorylation of Ser 473 and Thr 308 in Akt in all these cell lines (Figure 4A,B). Phosphorylation in Ser 65 in the eukaryotic translation initiation factor 4E-binding protein 1 (p-Ser 65-4E-BP1), a member of the family of translation repressor proteins and a well-known substrate of mechanistic target of rapamycin (mTOR), was also inhibited in all cell lines (Figure 4C). Western blot analysis confirmed the in-Cell ELISA measurements (Figure 4D). The data suggests that the Akt/mTOR pathway is inhibited by ATX-101 treatment. When classifying the 12 GBM cell lines used in this study by their p-Ser 473 Akt status (Supplementary Table S1, High or Low), and comparing this with their IC50 values (Figure 1A), a correlation (*p* = 0.03) between high sensitivity for ATX-101 and high p-Ser 473 Akt status was detected (Supplementary Figure S1C).

### 3.4. ATX-101 Reduces Stemness of GICs

**Figure 5:**
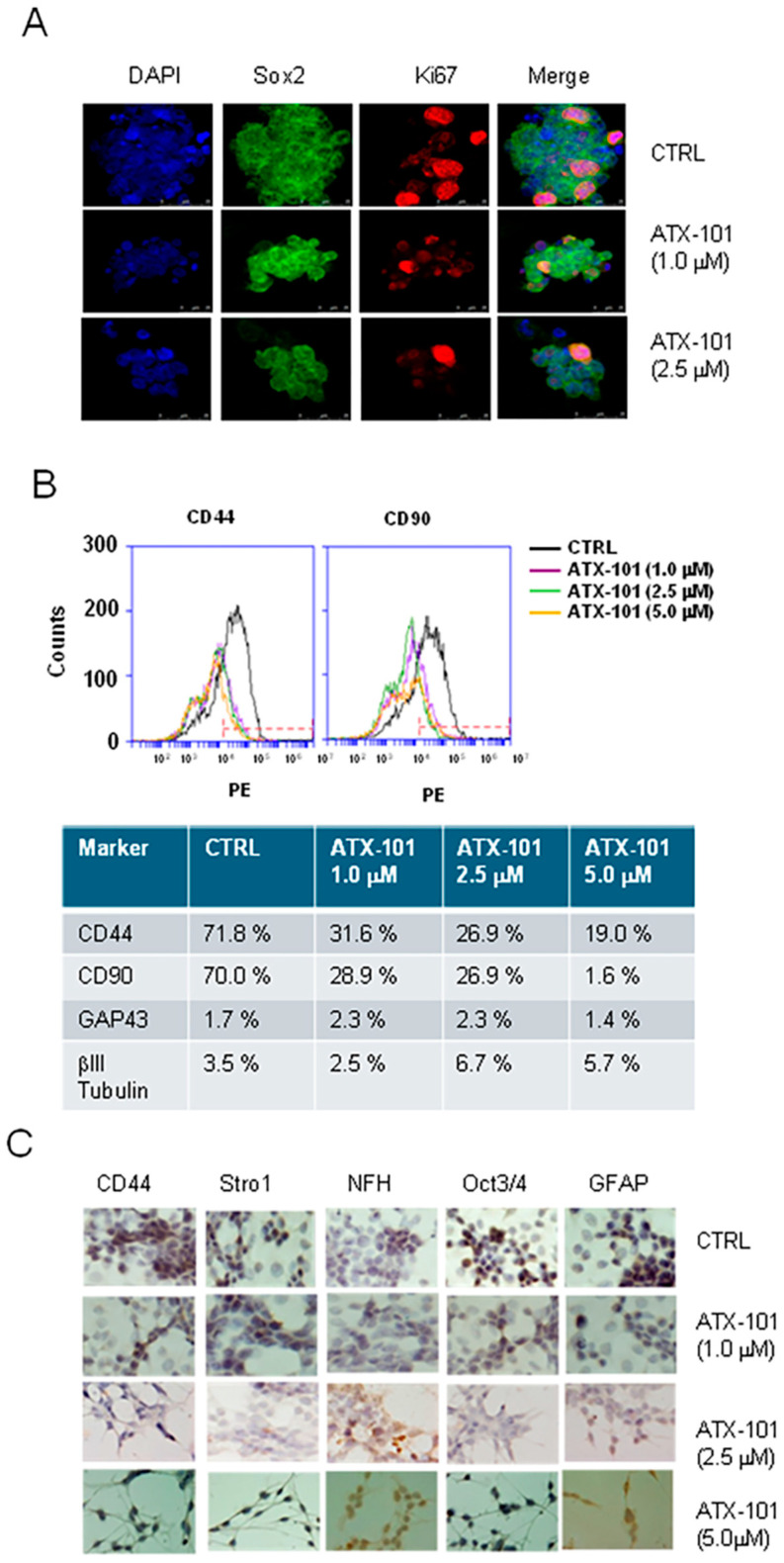
ATX-101 inhibits stemness phenotype and induces a reversion of Neural/proneural to mesenchymal phenotype. (**A**) Confocal analyses of Ki67- and Sox2-stained GSCs-5 cells treated with ATX-101 (1.0 and 2.5 μM) for 48 h. Bar indicates 25 μm. (**B**) FACS analyses for mesenchymal markers CD44 and CD90 in GSCs-5 cells after treatment with ATX-101 (1.0, 2.5, and 5 μM) for 48 h. Percentages of cells positive for CD44, CD90, GAP43, and βIII tubulin after treatment with ATX-101 are summarized in the table below the histograms. (**C**) ICC analyses performed on GSCs-5 cells for CD44, Stro1, NFH, OCT3/4, and GFAP after treatment with ATX-101 (1.0, 2.5, and 5 μM) for 48 h. Bar indicates 10 μm.

It has been demonstrated that the activity of Akt/TORC1 pathways modulates the stemness of several cancer stem cells in vitro including GBM [55,65,66,67,68]. We have previously observed that the dual TORC1/TORC2 inhibitor, RES529, affected the differentiation status of GICs [55]; thus, in the next step, we examined the effects of ATX-101 on stemness. ATX-101 reduced the expression of Ki67 and Sox2 in GSCs-5 cells, thus reducing both cell proliferation and stemness (Figure 5A). FACS analysis showed that the percentage of mesenchymal markers, CD44 and CD90, was reduced by ATX-101 treatment in a dose-dependent manner, while the fractions of cells positive for neural markers, GAP43 and βIII tubulin, were unchanged (Figure 5B, quantification in lower panel). Next, we extended the analysis on expression of mesenchymal (CD44 and Stro1), neural (NFH), stem (OCT3/4), and glial (GFAP) markers in ATX-101-treated GSCs-5 cells by ICC analyses (Figure 5C). These analyses showed that the expression of mesenchymal and stem cell markers was dose-dependently reduced by ATX-101 treatment, while NFH was significantly increased and that fewer cells were stained for GFAP. This suggests an increased glial differentiation after treatment with ATX-101. Altogether, these results suggest a partial reversion of the proneural to mesenchymal transition characteristic of GICs, equivalent to the epithelial-to-mesenchymal transition (EMT) phenomenon for epithelial cancers [55,69].

### 3.5. ATX-101 Has Radiosensitizing Effects

**Figure 6:**
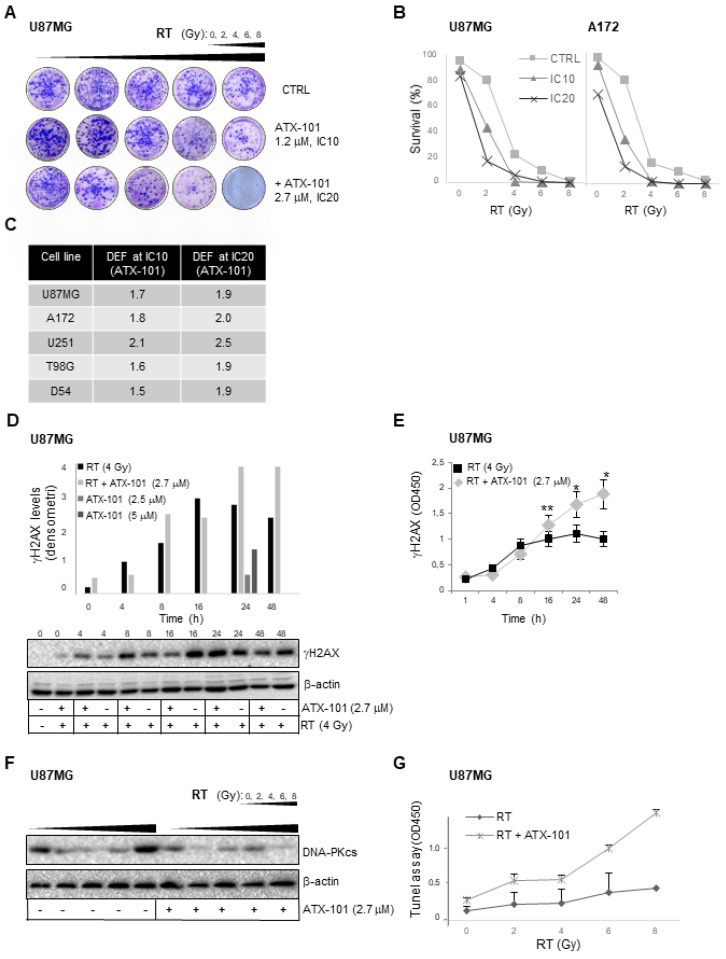
ATX-101 has radiosensitizing effects. (**A**) Representative images of crystal-violet-stained U87MG colonies (clonogenic assay) treated with ATX-101 IC10 (1.2 μM) and IC20 (2.7 μM) in combination with RT (2, 4, 6, and 8 Gy). (**B**) Surviving fractions of U87MG and A172 cells from clonogenic assays after treatment of cells with ATX-101 IC10 (1.2 μM) and IC20 (2.7 μM) in combination with RT (2, 4, 6, and 8 Gy). (**C**) Drug enhancement factor (DEF) for IC10 and IC20 doses of ATX-101 calculated in U87MG, A172, U251, T98G, and D54 cells. (**D**) Western blots showing γH2AX levels in U87MG treated with RT (4 Gy) alone or in combination with ATX-101 IC20 (2.7 μM) for 0–48 h (lower panel). Graphical presentation of densitometric values from the Western blots after normalization against β-actin (upper panel). Normalized values of γH2AX in ATX-101 single treated U87MG cells (2.5 and 5 μM) are shown for 24 h only. (**E**) γH2AX levels determined by in-Cell ELISA (OD450) from U87MG treated with RT (4 Gy) alone or in combination with ATX-101 IC20 (2.7 μM) for 24 h, ** *p* < 0.01, * *p* = 0.05. (**F**) Western blots showing the levels of activated DNA-PKcs in U87MG cells treated with ATX-101 IC20 (2.7 μM) in combination with RT (2, 4, 6, and 8 Gy) for 24 h. Uncropped Western blots in (**D**,**F**) are shown in Supplementary Figure S6. (**G**) TUNEL assay in U87MG cells treated with ATX-101 IC20 (2.7 μM) in combination with RT (2, 4, 6, and 8 Gy) for 24 h.

Because multiple proteins involved in DNA repair and damage tolerance must interact with PCNA in order to be fully active [34,35,37,63,64], and because ATX-101 is shown to increase the activity of multiple DNA-damaging agents [32,35,38,40,41,42,43], we next examined if ATX-101 affected the activity of RT. Combination experiments between RT and experimental drugs are commonly conducted by using IC10 and IC20 doses of the drug combined with different doses of RT. IC10 and IC20 of ATX-101 were determined for the different cell lines (Supplementary Figure S1A,D) and combined with increasing doses of RT (2–8 Gy). Images of stained U87MG colonies and graphic presentations of survival fractions of U87MG and A172 at increasing RT combined with IC10 and IC20 doses of ATX-101 are shown in Figure 6A,B, respectively. ATX-101 enhanced the effect of RT in both cell lines (Figure 6B), and the drug enhancement factor (DEF) shows that this also was the case in the U251, T98G, and D54 cell lines (Figure 6C). DEFs between 1.5 and 2.5 were calculated, with higher DEF values for IC20 compared to IC10 doses of ATX-101. Therefore, ATX-101 showed a dose-dependent radiosensitizing effect in all cell lines tested.

Because RT induces DNA damage, we examined the changes in γH2AX levels after cotreatment of GBM cells with the IC20 dose of ATX-101 and 4 Gy. An increased level of γH2AX was found in the combination-treated cells after 24 h (Figure 6D, graphic presentation in upper panel and Western blot in lower panel), suggesting increased levels of DNA double-strand breaks (DSBs). ATX-101, as a single-agent treatment at 2.5 and 5 μM, resulted in much lower levels of γH2AX than RT alone and the combination treatment (Figure 6D, shown at 24 h in graph, upper panel). Increased γH2AX in U87MG cells 24 h after treatments with the RT + ATX-101 combination was verified by in-Cell ELISA assays (Figure 6E). A comparable effect was detected in T98G and A172 cell lines treated with the same RT + ATX-101 combination by in-Cell ELISA (Supplementary Figure S5).

To further explore the DNA damage responses to DSBs, we measured activation of the DNA-dependent serine/threonine-protein kinase DNA-PKcs in U87MG cells treated for 8 h with RT alone or in combination with ATX-101. We found that ATX-101 reduced activation of DNA-PKcs (Figure 6F). Because DNA-PKcs is required for the nonhomologous end-joining (NHEJ) pathway of DSBs repair, this suggests that ATX-101 reduces these cells’ ability to repair DSBs. This agrees with the increased γH2AX levels detected (Figure 6D,E). Further, measurement of apoptosis via DNA fragmentation using the TUNEL assay supports that ATX-101 sensitized U87MG cells for RT as increased apoptotic cell death was detected after 24 h at several doses of RT (Figure 6G). This further supports reduced DNA repair and increased levels of DSBs in combination-treated cells relative to RT alone.

### 3.6. ATX-101 Inhibits Tumor Growth and Increases RT Efficacy in Nude Mice Bearing U87MG and T98G Subcutaneous Xenografts

**Figure 7:**
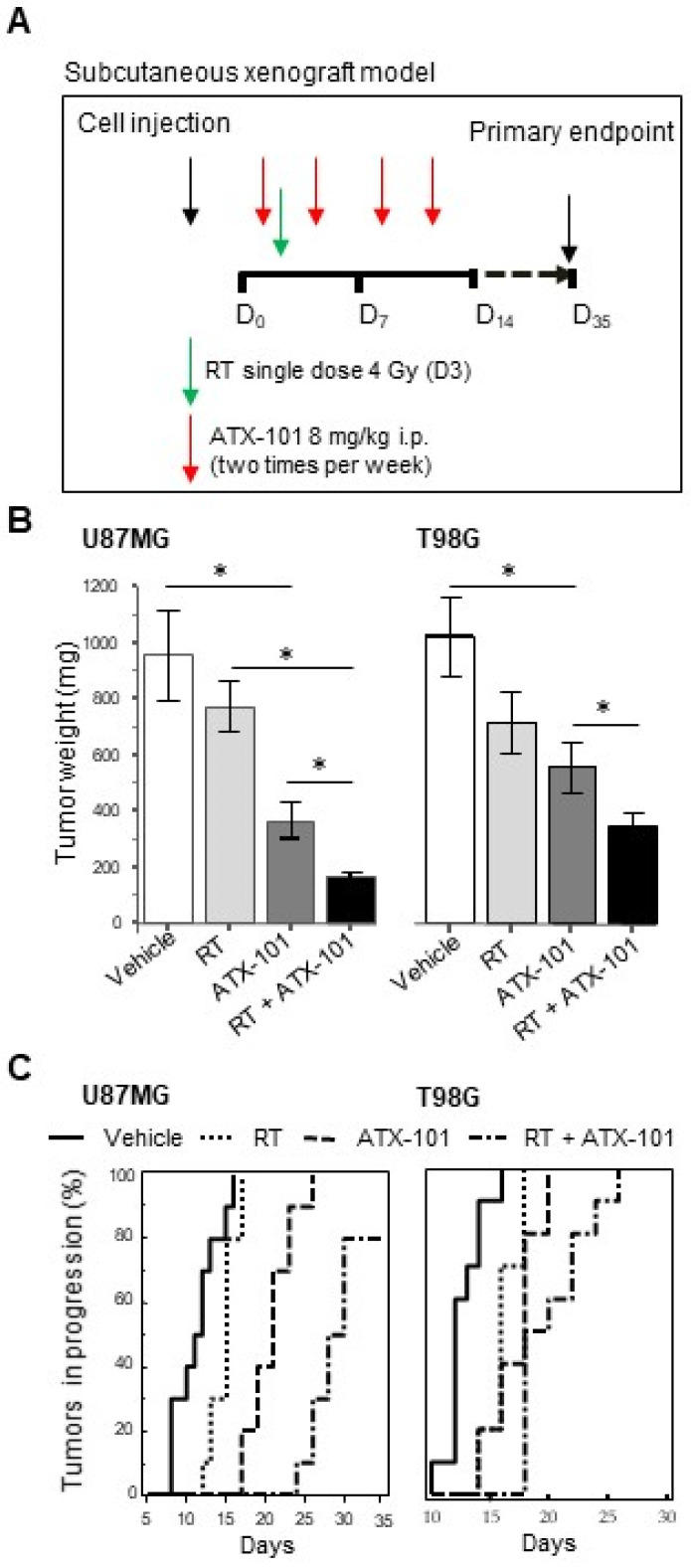
ATX-101 reduces the growth of subcutaneous xenograft GBM tumors. (**A**) Treatment scheme: 5 animals in each group received subcutaneous flank injections (2 tumors per mice). Vehicle or ATX-101 (8 mg/kg) were administered intraperitoneally (i.p.) two times per week (D2, D5, D9, D12, D16, D19, D23, D26, D30, and D33); radiotherapy (RT) (4 Gy) was given as a single administration on D3. (**B**) Weight of tumors collected from mice injected with U87MG (MGMT negative cells) and T98G cells (MGMT-positive tumor cells) at day 35 after treatment start. * *p* < 0.01. (**C**) Percentage of tumors in progression/Kaplan–Meier curves for the same xenografts as in B. Statistics are shown in Supplementary Table S3.

For initial in vivo efficacy tests of ATX-101, alone and in combination with RT, a subcutaneous xenograft model and the treatment regimen shown in Figure 7A were used. The dose of ATX-101 was selected based on previous animal experiments [40,41]. Treatment with ATX-101 as a single treatment reduced the tumor weight by 61% for U87MG and 35% for T98G xenografts compared to tumors in untreated animals (Figure 7B, Supplementary Table S2). Because no correlation between IC50 values and MGMT, PTEN, or p53 status was detected analyzing five MGMT positive and seven MGMT negative cell lines (Supplementary Figure S1C and Supplementary Table S1), the different responses to ATX-101 in the U87MG and T98G xenografts are likely due to other causes than their MGMT status. These other causes do not include their Akt status, as both cell lines are characterized by high p-Ser 373-Akt (Supplementary Table S1). RT as a single treatment at the dose used was less efficient than ATX-101 in both models, but a significant combinatory effect was observed.

When plotting the percentage of tumors in progression versus time, generating Kaplan–Meier curves, a clear combinatory effect of ATX-101 and RT was seen in both models (Figure 7C, statistics in Supplementary Table S3).

### 3.7. ATX-101 Reduced the Growth of Intrabrain Tumors Originated from Luciferase-Tagged U87MG and GSCs-5 Cells

**Figure 8:**
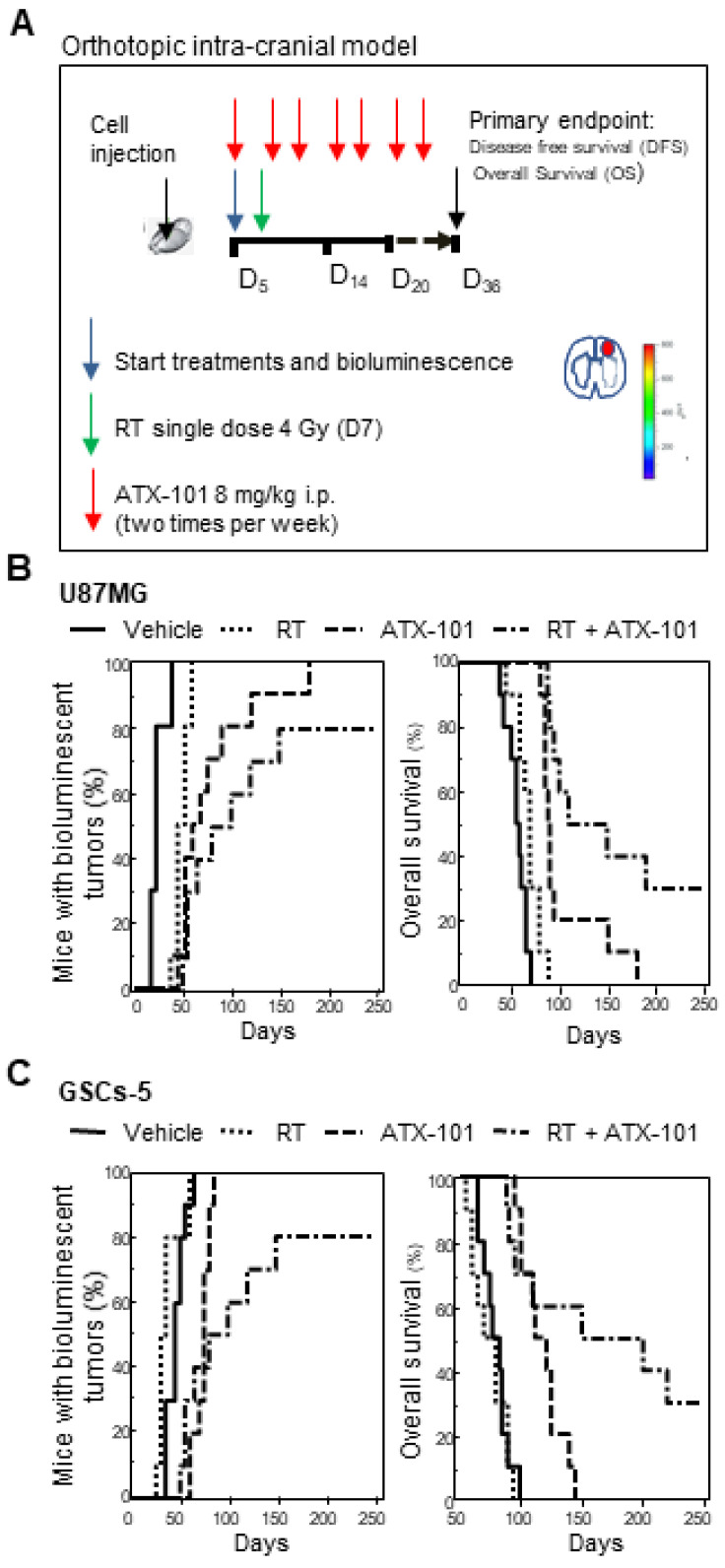
ATX-101 reduces the growth of intrabrain U87MG and GSCs-5 tumors. (**A**) Treatment scheme: 10 animals in each group received intrabrain injection of 1 × 10^3^ luciferase-tagged U87MG or GSCs-5 cells. Vehicle or ATX-101 (8 mg/kg) were administered intraperitoneally (i.p.) two times per week (D5, D8, D12, D15, D19, D22, D26, D29, and D33); radiotherapy (RT) (4 Gy) was given as a single administration on D7. Primary endpoint was set at D36, but the mice were followed for up to 250 days. (**B**,**C**) Kaplan–Meier curves of percentage of mice with bioluminescent tumors, i.e., mice with progression, and overall survival, U87MG (**B**), and GSCs-5 (**C**). Statistics are shown in Tables S4 and S5.

It has been demonstrated that ATX-101 is able to penetrate the blood–brain barrier in rats [41]; thus, the efficacy of ATX-101 in a murine intrabrain tumor model was subsequently examined. A small number of luciferase-positive U87MG and GSCs-5 cells were inoculated into the brain to mimic a clinical situation where a small residual mass may remain after surgery. Animals with brain tumors were treated over 33 days with a follow-up of 250 days without drug administration (treatment regimen is shown in Figure 8A). As observed in the xenograft models, ATX-101 as a single agent had better efficacy than RT alone in both intracranial models, and the combination treatment was superior to both single treatments (Figure 8B,C, statistics in Supplementary Tables S4 and S5 for U87MG and GSCs-5, respectively).

Vehicle-treated mice with U87MG cells developed bioluminescent intrabrain tumors between 15 and 35 days from the start, resulting in a calculated disease-free survival (DFS) of 21.5 ± 2.4 days (mean ± SEM). DFS increased to 114.5 ± 24.7 days in the combination group, and 2 out of 10 animals showed no tumor at the end of the study (for DFS and statistics see Supplementary Table S4). Mean overall survival (OS) in the combination group was 157.3 ± 22.55 days with a range of 88–250 days, and 3 mice were still alive on Day 250 (Figure 8B, Supplementary Table S4A).

Vehicle-treated mice with GSCs-5 cells developed bioluminescent intrabrain tumors between 35 and 65 days after treatment start with a DFS of 46.0 ± 3.0 days (Supplementary Table S5A). DFS increased significantly after treatment with the combination of RT and ATX-101 to 117.5 ± 24.3 days, and 2 out 10 mice showed no tumors after 250 days (Figure 8C and statistics in Supplementary Table S5).

The combination index (CI) was calculated for the treatment effects in both models (Supplementary Tables S4 and S5). A synergistic effect for the combination of ATX-101 and RT was seen for OS in the GCSs-5 model, while additive effects were seen for OS in the U87MG model and for DFS in both models.

## 4. Discussion

One of the main therapeutic modalities for treating and controlling GBM is RT. However, the adaptive radioresistance of the tumor eventually leads to the failure of RT with a lethal progression of the disease. New active compounds that can be administered alone or in combination with RT are therefore urgently needed. Here, we show that the experimental drug ATX-101, for which a clinical phase I study in advanced solid tumor patients was recently finished [44], could be a promising new drug that enhances the efficacy and possible duration of responses to RT. Previously, ATX-101 was shown to enhance the efficacies of multiple anticancer drugs and resensitize cisplatin-resistant bladder cancer cells [32,40,41,42,43].

The sensitivities (IC50s) for ATX-101 varied from 4.3 to 10.2 μM in the different GBM and GIC cell lines; however, there was no correlation between sensitivity and PCNA levels, or p53, MGMT, and PTEN status. This suggests that ATX-101 may be effective on multiple GBM tumors independently of their genetic background. Until now, no single factor predicting ATX-101 sensitivity has been found, but GBM cell lines with high activation of Akt tend to be more sensitive. The lack of one predictive factor is likely due to the multiple functions of the target of ATX-101, PCNA. PCNA plays a central role as a scaffold protein both in the nucleus, directly interacting with DNA replication, DNA repair and damage tolerance, and in the cytosol, interacting with proteins involved in regulation of cellular signaling and apoptosis [32,33,35,40,41,42,43,63]. Thus, multiple stress-related conditions can lead to ATX-101 sensitivity. Here, we show that both cytosolic and nuclear roles of PCNA are affected; ATX-101 as a single agent induces apoptosis, stalls cancer cells in G2/M, reduces Akt/mTOR signaling and stemness of GICs, and when combined with RT, increases the level of γH2AX and DNA fragmentation and reduces the DNA damage response via reduction of DNA-PKcs. ATX-101 as a single agent did not increase γH2AX levels, supporting that the antitumor effect of ATX-101 alone is not mediated by DNA damage. This is in agreement with previous data showing that ATX-101 does not affect replication [32,40] and increases the efficacy of kinase inhibitors in the absence of DNA-damaging drugs [43]. In addition, ATX-101 impairs the ability to repair and/or tolerate DNA lesions and thereby increases the levels of DNA lesions introduced by DNA-damaging drugs [35,40,41,42].

Experiments have suggested that a small subpopulation of highly tumorigenic cells with stem cell properties (GICs) play a significant role in GBM aggression. These cells originate from intratumor areas with necrosis, a status indicative of hypoxia and extremely poor oxygen supply, which renders these cells resistant to RT and chemotherapy. These factors are believed to be important for their regrowth. We demonstrated here that ATX-101 reduced the stem cell phenotype of GSCs-5, i.e., reduced Sox2, CD44, CD90, and OCT3/4 expression. Further, ATX-101 inhibited proliferation and formation of neurospheres and induced apoptosis in these cells. Maintenance of stem cells and EMT phenotypes is regulated by the PI3K/Akt/mTOR pathway [65]; thus, the observed effects could be via ATX-101’s effect on this pathway. Altogether, these results suggest a partial reversion of the neural to mesenchymal transition characteristics of GICs (a phenomenon such as EMT for epithelial cancers) after treatment with ATX-101, a trait that might be important for use in GBM.

## 5. Conclusions

In this report, we demonstrated that ATX-101 has good activity against GBM in both in vitro and in vivo experiments. Importantly, ATX-101 was shown to reduce tumor growth in several animal models, including an intracranial tumor model, and potentiate the efficacy of RT. The mode of action of ATX-101 was verified to include dysregulation of cellular signaling and apoptosis both in the absence and presence of DNA damage via inhibition of both nuclear and cytosolic roles of PCNA. These results warrant further studies of ATX-101 for use in GBM therapies.

## Data Availability

The data presented in this study are available on request from the corresponding author.

## Supplementary Materials

The following supporting information can be downloaded at: https://www.mdpi.com/article/10.3390/cancers14020289/s1, Figure S1: Cell death induced by ATX-101 is independent of P53, PTEN and MGMT status in glioblastoma cell lines; Figure S2: Uncropped Western blots shown in Figure 1C; Figure S3: Uncropped Western blots shown in Figure 2A,B; Figure S4: Uncropped Western blots shown in Figure 4D; Figure S5: ATX-101 increases the levels of γH2AX induced by RT; Figure S6: Uncropped western blots shown in Figure 6D,F; Table S1: Characterization of GBM cell lines used; Table S2: Weights of mice and tumours in the subcutaneous xenograft model; Table S3: Radio-sensitization of ATX-101 on U87MG and T98G subcutaneous xenografts; Table S4: Statistical analyses for disease-free survival (DFS) and overall survival OS (A) and HR ratios (B) for animals treated with ATX-101 with or without RT for the orthotopic brain tumour model using luciferase tagged U87MG cells; Table S5: Statistical analyses for DFS and OS (A) and HR ratios (B) for animals treated with ATX-101 with or without RT for the orthotopic brain tumour model using luciferase tagged GSGs-5 cells.
