# Correction: Gravina et al. ATX-101, a Peptide Targeting PCNA, Has Antitumor Efficacy Alone or in Combination with Radiotherapy in Murine Models of Human Glioblastoma. *Cancers* 2022, *14*, 289

**DOI:** 10.3390/cancers16162897

**Published:** 2024-08-21

**Authors:** Giovanni Luca Gravina, Alessandro Colapietro, Andrea Mancini, Alessandra Rossetti, Stefano Martellucci, Luca Ventura, Martina Di Franco, Francesco Marampon, Vincenzo Mattei, Leda Assunta Biordi, Marit Otterlei, Claudio Festuccia

**Affiliations:** 1 Department of Biotechnological and Applied Clinical Sciences, Division of Radiation Oncology, University of L’Aquila, 67100 L’Aquila, Italy; giovanniluca.gravina@univaq.it; 2 Department of Biotechnological and Applied Clinical Sciences, Laboratory of Radiobiology, University of L’Aquila, 67100 L’Aquila, Italy; alecolapietro@gmail.com (A.C.); mancio_1982@hotmail.com (A.M.); alessandra.rossetti@graduateunivaq.it (A.R.); 3 Department of Biotechnological and Applied Clinical Sciences, Laboratory of Cellular Pathology, University of L’Aquila, 67100 L’Aquila, Italy; s.martellucci@sabinauniversitas.it; 4 Biomedicine and Advanced Technologies Rieti Center, Sabina Universitas, 02100 Rieti, Italy; vincenzo.mattei@uniroma1.it; 5 Division of Pathology, San Salvatore Hospital, 67100 L’Aquila, Italy; lventura@asl1abruzzo.it (L.V.); mdifranco@asl1abruzzo.it (M.D.F.); 6 Department of Radiological, Oncological and Pathological Sciences, Sapienza University of Rome, 00100 Rome, Italy; francesco.marampon@uniroma1.it; 7 Department of Biotechnological and Applied Clinical Sciences, Laboratory of Medical Oncology, University of L’Aquila, 67100 L’Aquila, Italy; leda.biordi@univaq.it; 8 APIM Therapeutics A/S, N-7100 Rissa, Norway; 9 Department of Clinical and Molecular Medicine, Norwegian University of Science and Technology (NTNU), N-7006 Trondheim, Norway

In the original publication [1], there was a mistake in Figure 5 (panel C) as published. This panel contained ICC images that were mistakenly sourced from experiments conducted on a different cell line. This error likely occurred when the pool of images, independently provided by several collaborators, was assembled. Unfortunately, it was not caught during the final internal review prior to submission. The new correct figure appears below. The text and figure legend are unchanged. The authors apologize for any inconvenience caused and state that the scientific conclusions are unaffected. This correction was approved by the Academic Editor.

## Figures and Tables

**Figure 5:**
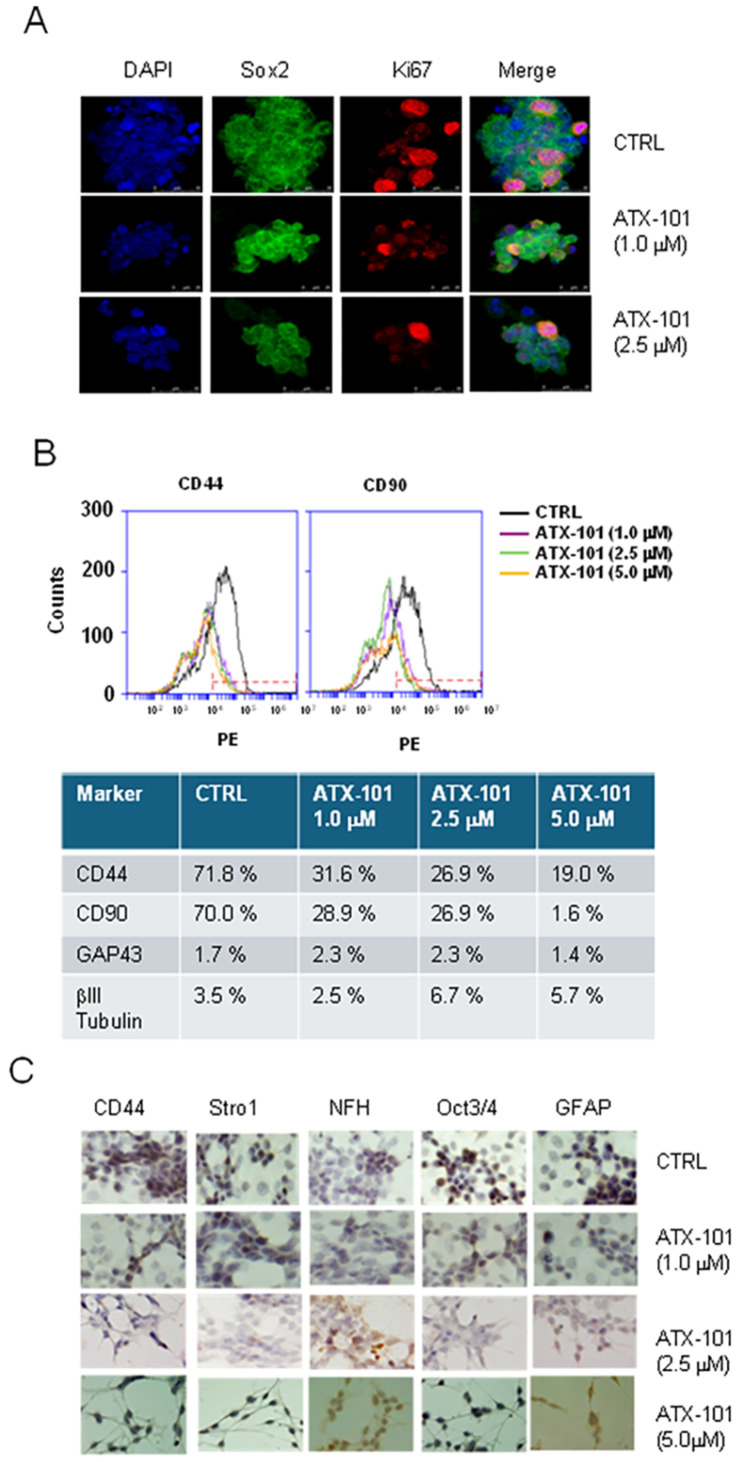
ATX-101 inhibits stemness phenotype and induces a reversion of Neural/proneural to mesenchymal phenotype. (**A**) Confocal analyses of Ki67- and Sox2-stained GSCs-5 cells treated with ATX-101 (1.0 and 2.5 μM) for 48 h. Bar indicates 25 μm. (**B**) FACS analyses for mesenchymal markers CD44 and CD90 in GSCs-5 cells after treatment with ATX-101 (1.0, 2.5, and 5 μM) for 48 h. Percentages of cells positive for CD44, CD90, GAP43, and βIII tubulin after treatment with ATX-101 are summarized in the table below the histograms. (**C**) ICC analyses performed on GSCs-5 cells for CD44, Stro1, NFH, OCT3/4, and GFAP after treatment with ATX-101 (1.0, 2.5, and 5 μM) for 48 h. Bar indicates 10 μm.

## References

[1] Gravina G.L., Colapietro A., Mancini A., Rossetti A., Martellucci S., Ventura L., Di Franco M., Marampon F., Mattei V., Biordi L.A. (2022). ATX-101, a Peptide Targeting PCNA, Has Antitumor Efficacy Alone or in Combination with Radiotherapy in Murine Models of Human Glioblastoma. Cancers.

