# Supplementary material for: ATX-101, a Peptide Targeting PCNA, Has Antitumor Efficacy Alone or in Combination with Radiotherapy in Murine Models of Human Glioblastoma
Source: Cancers (Basel). 2022 Jan 7;14(2):289. doi: 10.3390/cancers14020289 (PMC8773508; doi:10.3390/cancers14020289)
Supplement: Supplementary file 1 [file cancers-14-00289-s001.zip › cancers-1522088-supplementary.pdf]

cancers

MDPI

Article

ATX-101, a Peptide Targeting PCNA, Has Antitumor Efficacy Alone or in Combination with Radiotherapy in Murine Models of Human Glioblastoma

GRAVINA et al, 2021.

Supplementary material

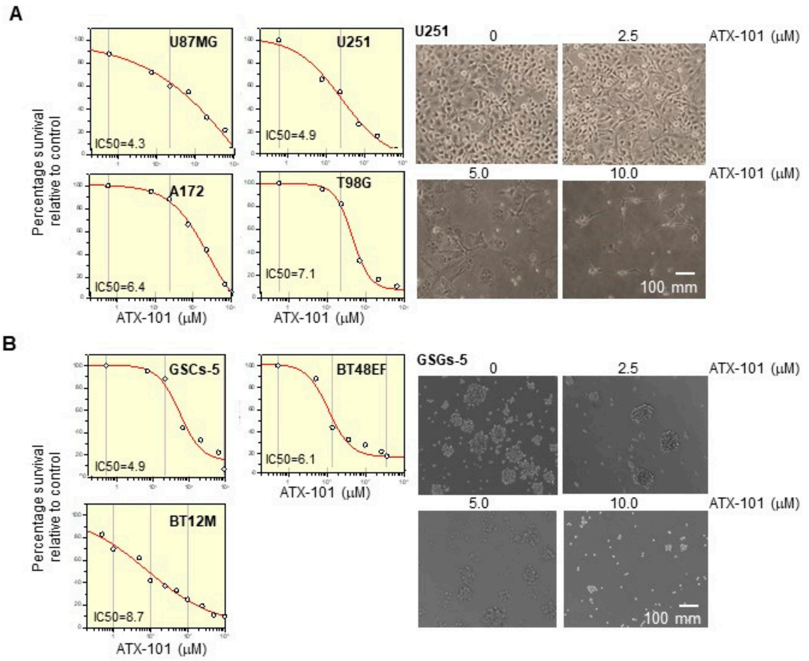

C

Comparisons	IC50 (μM ± SEM)	Statistics
p53 WT vs p53 loss	5.9 ± 1.3 vs 6.2 ± 0.7	p=0.9 (NS)
p53 WT vs gain of function	6.0 ± 0.5 vs 6.9 ± 1.5	p=0.6 (NS)
PTEN + vs PTEN -	6.0 ± 0.8 vs 5.8± 0.4	p=0.8 (NS)
MGMT + vs MGMT -	7.5 ± 1.7 vs 5.1 ± 0.4	p=0.08 (NS)
GBM vs GICs	1.6 ± 0.4 vs 3.4 ± 0.8	p=0.06 (NS)
High vs Low p-Ser 473-Akt expression	5.0 ± 0.2 vs 8.4 ± 0.7	p=0.003

D

Cell lines	IC20 (μM ± SD)	IC10 (μM ± SD
GBM cell lines		
U87MG	2.7 ± 0.5	2.0 ± 0.2
U251	2.0 ± 0.2	0.8 ± 0.1
A172	2.6 ± 0.4	1.8 ± 0.2
T98G	3.2 ± 0.2	2.2 ± 0.2
U373	2.0 ± 0.4	0.6 ± 0.2
U118	2.6 ± 0.4	2.0 ± 0.3
D54	4.9 ± 0.7	3.7 ± 0.5
SW1783	2.7 ± 0.3	1.9 ± 0.3
LN229	2.4 ± 0.2	1.4 ± 0.2
U138	4.3 ± 0.5	2.8 ± 0.3
SF268	2.3 ± 0.2	1.3 ± 0.2
SNB19	5.7 ± 0.7	4.8 ± 0.5
Patient derived GICs		
BT12M	5.2 ± 0.7	4.3 ± 0.5
BT48EF	3.7 ± 0.5	1.8 ± 0.2
BT50EF	2.3 ± 0.2	1.3 ± 0.2
GSCs-5	2.4 ± 0.3	1.2 ± 0.1
GSCs-7	3.2 ± 0.5	2.5 ± 0.6

Figure S1. Cell death induced by ATX-101 is independent of P53, PTEN and MGMT status in glioblastoma cell lines. (A) Representative inhibition curves for ATX-101 treatment of U87MG, U251, A172 and T98G GMB cell lines (left panel). Representative images from U251 cell culture treated with different doses of ATX-101 (right panel). (**B**) Representative inhibition curves for ATX-101 treatment of the GICs GSCs-5, BT48EF and BT12M. Morphological appearance of GSCs-5 cultures treated with different doses of ATX-101 (right panel) (**C**) Comparisons between p53, PTEN, MGMT and p-Ser 473 Akt expression levels/status and IC50 values. Comparisons between IC50 values of GBM vs GIC cell lines. (**D**) IC20 and IC10 values calculated from survival curves. NS= not significant.

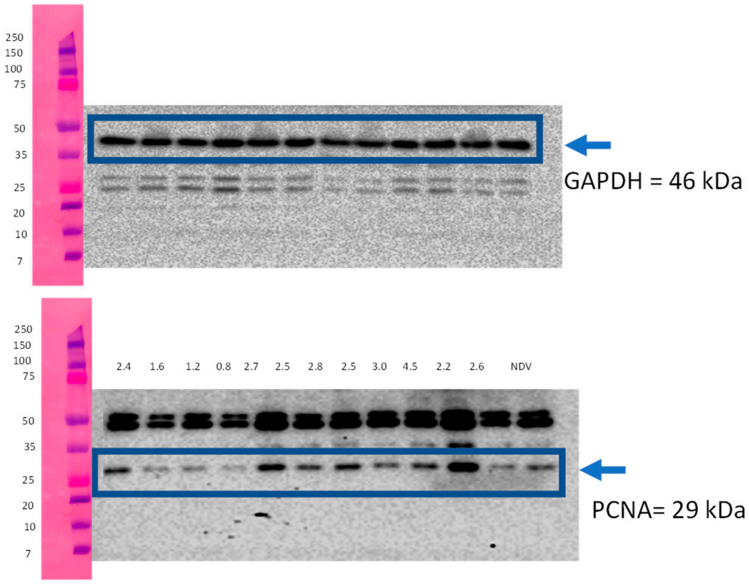

Figure S2. Uncropped Western blots shown in Figure 1C. For detailed description see figure legend in main manuscript.

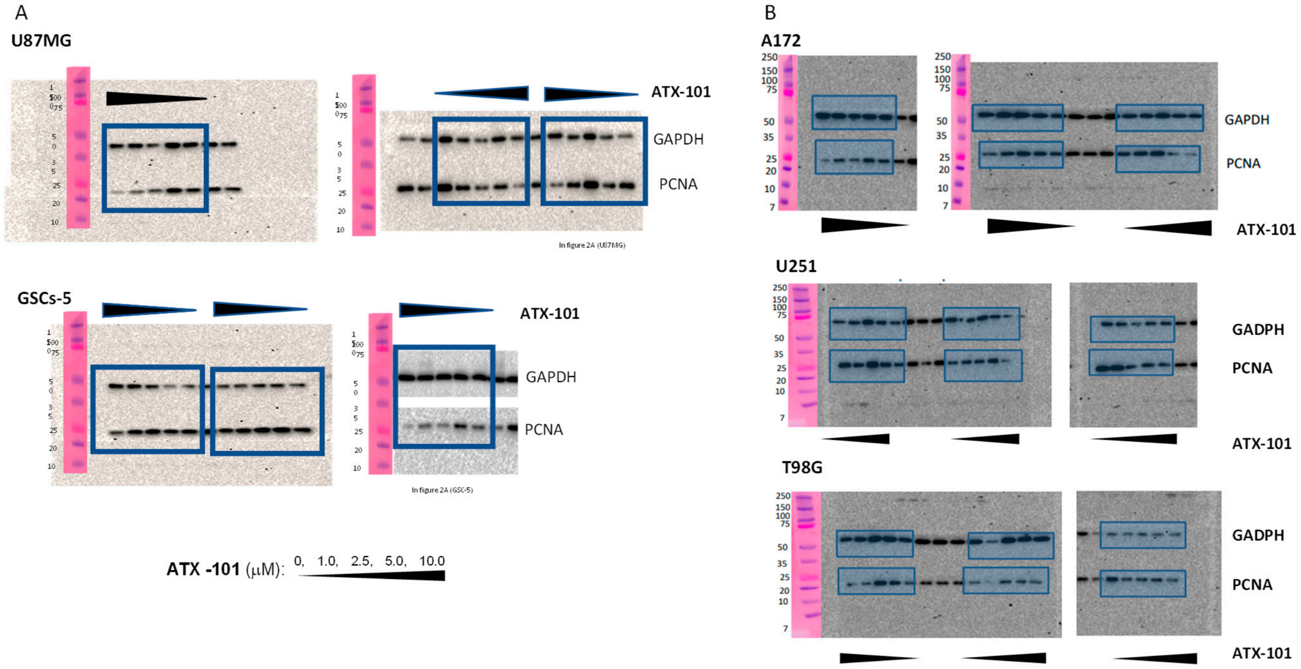

Figure S3. Uncropped Western blots shown in Figure 2A and B. For detailed description see figure legend in main manuscript.

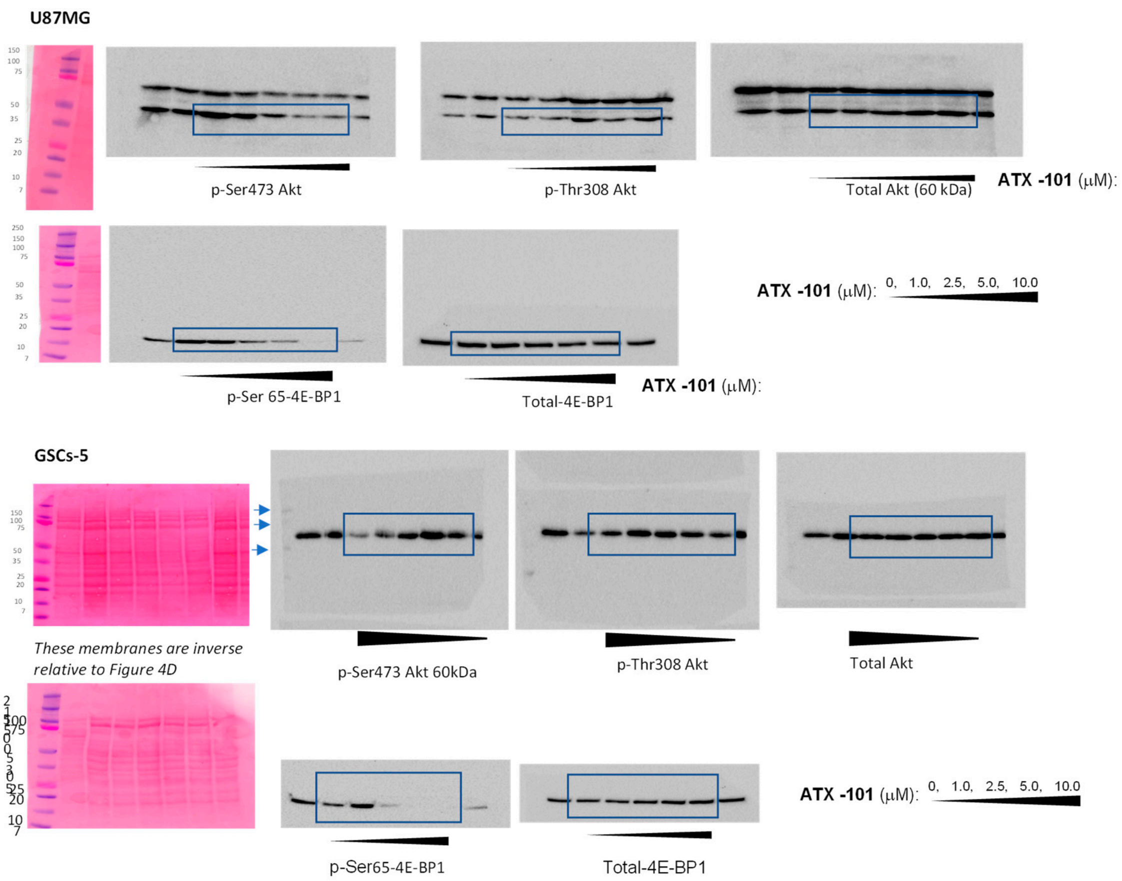

Figure S4. Uncropped Western blots shown in Figure 4D. For detailed description see figure legend in main manuscript.

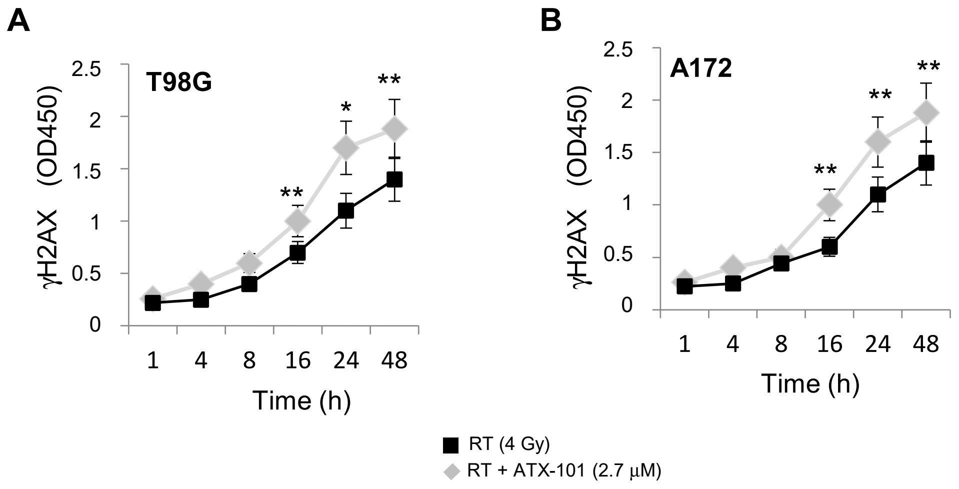

Figure S5. ATX-101 increases the levels of γH2AX induced by RT. ELISA detection for γH2AX in (**A**) T98G and (**B**) A172 glioma cell lines treated with ATX-101 (2.7 µM) and RT (4 Gy) for 1- 48 h. Mean values of data from 3 cell extracts analysed in triplicates (total 9 determinations) ± SD are shown. *p<0.05, ** p<0.01.

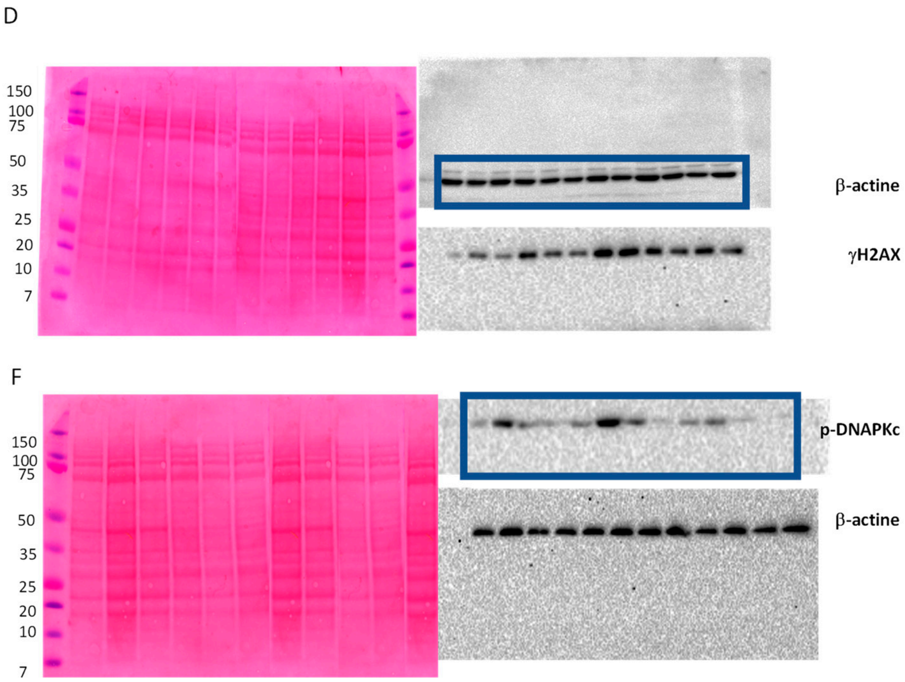

Figure S6. Uncropped Western blots shown in Figure 6D and F. For detailed description see figure legend in main manuscript.

Table S1. Characterization of GBM cell lines used.

Cell line	MGMT	TMZ	PTEN	p53	p-Ser 373-Akt	RT
U87MG	Methylated (-)	S	PTEN/mutated	p53 (+ WT)	High	S
U251	Methylated (-)	S	PTEN/null	p53/mutated	High	S
T98G	Non-methylated (+)	R	PTEN/null	p53/mutated	High	Moderately
A172	Methylated (-)	S	PTEN (+WT)	p53 (+WT)	Low	Moderately
D54	Methylated (-)	S	PTEN (+WT)	p53 (+WT)	Low	R
SW1783	Methylated (-)	S	PTEN (+WT)	p53 (+WT)	Low	R
U118	Non-methylated (+)	R	PTEN/null	p53 (+WT)	High	R
U373	Methylated (-)	S	PTEN/null	p53/mutated	High	S
SNB19	Non-methylated (+)	R	PTEN mutated	p53 (+WT)	High	Moderately
L229	Methylated (-)	S	PTEN (+WT)	p53/mutated	Low	S
SF268	Non-methylated (+)	R	PTEN/mutated	p53/mutated	High	Moderately
U138	Non-methylated (+)	R	PTEN (+WT)	p53/mutated	low	R

Table S2. Weights of mice and tumours in the subcutaneous xenograft model.
Data shown in Figure 7B.

Cells, Treatments	Weight of mice (mean ± SEM)	Tumour weight (mean ± SEM)
U87MG, Vehicle	25.0 ± 0.3	987 ± 71
U87MG, ATX-101	25.0 ± 0.2	385 ± 55
U87MG, RT	24.8 ± 0.5	775 ± 40
U87MG, RT + ATX-101	24.5 ± 0.8	166 ± 24
T98G, Vehicle	24.5 ± 0.3	1015 ± 61
T98G, ATX-101	26.0 ± 0.2	655 ± 40
T98G, RT	24.8 ± 0.5	710 ± 46
T98G, RT + ATX-101	24.5 ± 0.3	346 ± 22

Table S3. Radio-sensitization of ATX-101 on U87MG and T98G subcutaneous xenografts. Statistical analyses of Kaplan Meyer curves shown in Figure 7.

	**U87MG**				**T98G**			
Comparisons	HR	95% CI	Statistics	Combination index, CI	HR	95% CI	Statistics	Combination index, CI
Vehicle vs ATX-101	4.3	1.5 - 15.4	p<0.001	ATX+RT CI=1.05 (Additive)	3.4	1.2 - 11.1	p<0.001	ATX+RT CI=0.93 (sub-additive)
Vehicle vs RT	2.2	1.0 - 6.6	p<0.05		3.0	1.0 - 9.7	p<0.001	
RT vs ATX-101	3.8	1.3 - 11.2	p<0.005		1.5	0.6 - 3.9	NS	
Vehicle vs ATX-101 + RT	6.8	1.9-- 21.1	p<0.0001		5.9	1.4 - 17.7	p<0.0001	
ATX-101 vs ATX-101 + RT	3.6	1.2 - 9.17	p<0.01		3.0	1.0 - 8.5	p<0.01	
RT vs ATX-101 + RT	4.6	1.5 - 14.2	p<0.001		2.3	0.9 - 6.4	p<0.05	

NS= not significant.

Table S4. Statistical analyses for disease-free survival (DFS) and overall survival OS (A) and HR ratios (B) for animals treated with ATX-101 with or without RT for the orthotopic brain tumour model using luciferase tagged U87MG cells. Kaplan Meyer curves shown in Figure 8.

(**A**)

	**Disease-free survival (DFS)**			**Overall survival (OS)**		
Treatment	Range (days)	Mean ± SEM	Statistics	Range (days)	Mean ± SEM	Statistics
Vehicle	15-35	21.5 ± 2.4		40-70	56.0 ± 3.0	
ATX-101	30-120	53.0 ± 8.8	p<0.01 vs Vehicle	80-180	102.7 ± 10.7	p<0.01 vs Vehicle
RT	25-40	330 ± 4.8	p<0.01 vs Vehicle<br>p<0.05 vs ATX-101	45-90	69.0 ± 4.0	p<0.05 vs Vehicle<br>p<0.01 vs ATX-101
ATX-101 + RT	50-250	114.5 ± 24.7	p<0.01 vs Vehicle<br>p<0.01 vs ATX-101<br>p<0.01 vs RT	88-250	157.3 ± 22.5	p<0.01 vs Vehicle<br>p<0.05 vs ATX-101<br>p<0.01 vs RT

(**B**)

	**Disease-free survival (DFS)**				**Overall survival (OS)**			
Comparison	HR	95% CI	Statistics	Combination index, CI	HR	95% CI	Statis-tics	Combination index, CI
Vehicle vs ATX-101	3.3	1.2-11.4	p<0.01	ATX+RT CI=1.02 Additive	4.9	1.5-15.1	p<0.01	ATX+RT CI=1.06 Additive
Vehicle vs RT	2.4	0.9-6.3	p<0.01		2.4	1.0-7. 0	p<0.01	
RT vs ATX-101	2.5	0.9-6.5	p<0.01		3.3	1.2-9.4	p<0.01	
Vehicle vs ATX-101 + RT	5.5	2.0-13.9	p<0.01		7.7	2.6-16.9	p<0.01	
ATX-101 vs ATX-101 + RT	4.9	1.5-15.0	p<0.01		4.0	1.4-10.0	p<0.01	
RT vs ATX-101 + RT	3.0	1.1-8.3	p<0.01		5.1	1.6-15.6	p<0.01	

Table S5. Statistical analyses for DFS and OS (A) and HR ratios (B) for animals treated with ATX-101 with or without RT for the orthotopic brain tumour model using luciferase tagged GSGs-5 cells. Kaplan Meyer curves shown in Figure 8.

(**A**)

	**Disease-free survival (DFS)**			**Overall survival (OS)**		
Treatment	Range (days)	Mean ± SEM	Statistics	Range (days)	Mean ± SEM	Statistics
Vehicle	35-65	46.0 ± 3.0		40-70	79.5 ± 3.6	
ATX-101	60-85	73.5 ± 2.6	p<0.001 vs Vehicle	94-145	74.5 ± 4.6	NS vs Vehicle
RT	25-60	37.0 ± 4.0	NS vs Vehicle<br>p<0.01 vs ATX-101	45-90	69.0 ± 4.0	p<0.05 vs Vehicle<br>p<0.01 vs ATX-101
ATX-101 + RT	50-250	117.5 ± 24.3	p<0.01 vs Vehicle<br>p<0.01 vs ATX-101<br>p<0.01 vs RT	88-250	170.3 ± 22.4	p<0.01 vs Vehicle<br>p<0.01 vs RT<br>p<0.01 vs ATX-101

NS= not significant.

(**B**)

<table>
<tr><th></th><th colspan="4">Disease-free survival (DFS)</th><th colspan="4">Overall survival (OS)</th></tr>
<tr><td>Comparison</td><td>HR</td><td>95% CI</td><td>Statis-tics</td><td>Combination index, CI</td><td>HR</td><td>95% CI</td><td>Statistics</td><td>Combination index, CI</td></tr>
<tr><td>Vehicle vs ATX-101</td><td>4.0</td><td>1.3-11.1</td><td>p<0.01</td><td rowspan="6">ATX+RT
CI=0.97
Sub-Additive</td><td>4.4</td><td>1.4-13.7</td><td>p<0.01</td><td rowspan="6">ATX+RT
CI=1.11
Synergistic</td></tr>
<tr><td>Vehicle vs RT</td><td>0.6</td><td>0.3-1.5</td><td>NS</td><td>0.8</td><td>0.3-2.0</td><td>NS</td></tr>
<tr><td>RT vs ATX-101</td><td>4.2</td><td>1.3-16.7</td><td>p<0.01</td><td>4.7</td><td>1.48-13.57</td><td>p<0.01</td></tr>
<tr><td>Vehicle vs ATX-101 + RT</td><td>4.5</td><td>1.3-11.4</td><td>p<0.01</td><td>5.8</td><td>1.6-14.9</td><td>p<0.01</td></tr>
<tr><td>ATX-101 vs ATX-101 + RT</td><td>1.9</td><td>0.7-4.9</td><td>NS</td><td>2.4</td><td>0.9-6.3</td><td>p<0.05</td></tr>
<tr><td>RT vs ATX-101 + RT</td><td>3.9</td><td>1.3-11.4</td><td>p<0.01</td><td>4.5</td><td>1.5-13.6</td><td>p<0.01</td></tr>
</table>

NS= not significant.
